# Statistical methodology for the evaluation of vaccine efficacy in a phase III multi-centre trial of the RTS,S/AS01 malaria vaccine in African children

**DOI:** 10.1186/1475-2875-10-222

**Published:** 2011-08-04

**Authors:** Marc Lievens, John J Aponte, John Williamson, Bruno Mmbando, Ali Mohamed, Philip Bejon, Amanda Leach

**Affiliations:** 1 GlaxoSmithKline Biologicals, Wavre, Belgium; 2 Centre de Recerca en Salut Internacional de Barcelona (CRESIB), Hospital Clínic, Universitat de Barcelona, Spain; 3 KEMRI/CDC Research and Public Health Collaboration, Kisumu, Kenya; 4 National Institute for Medical Research, Dar es salaam, Tanzania; 5 Ifakara Health Institute, Ifakara, Tanzania; 6 KEMRI-Wellcome Trust Research Program, Kilifi, Kenya

## Abstract

**Background:**

There has been much debate about the appropriate statistical methodology for the evaluation of malaria field studies and the challenges in interpreting data arising from these trials.

**Methods:**

The present paper describes, for a pivotal phase III efficacy of the RTS, S/AS01 malaria vaccine, the methods of the statistical analysis and the rationale for their selection. The methods used to estimate efficacy of the primary course of vaccination, and of a booster dose, in preventing clinical episodes of uncomplicated and severe malaria, and to determine the duration of protection, are described. The interpretation of various measures of efficacy in terms of the potential public health impact of the vaccine is discussed.

**Conclusions:**

The methodology selected to analyse the clinical trial must be scientifically sound, acceptable to regulatory authorities and meaningful to those responsible for malaria control and public health policy.

**Trial registration:**

Clinicaltrials.gov NCT00866619

## Background

In May 2009, the first large scale, phase III efficacy trial assessing a candidate malaria vaccine was launched across seven countries in sub-Saharan Africa. The vaccine candidate RTS, S/AS01 targets the pre-erythrocytic stage of the *Plasmodium falciparum *parasite. This phase III trial is designed to determine the efficacy of the vaccine against clinical malaria in young children to support the file submission for regulatory review by European and African regulatory authorities. Additionally, a broad range of secondary efficacy endpoints are included to evaluate the full potential direct impact of the vaccine on child health. These include severe malaria and malaria-specific mortality as well as all-cause hospitalization and mortality.

Malaria is a common disease in sub-Saharan children and so it may at first appear relatively simple to design trials to demonstrate the effects of interventions with reasonable sample sizes. However, the design of such trials comes with specific statistical challenges. All individuals are not at the same level or risk of malaria disease; transmission varies widely across trial sites. Malaria control measures are being scaled-up across sub-Saharan Africa, but at differing rates and coverage. Statistical methods must be able to adjust for the confounding effect of explanatory variables and take into account the fact that multiple events within the same individual are not independent.

Phase II data suggest that while RTS, S/AS01 offers approximately 50% protection against clinical malaria disease, the likely mechanism of protection is reduction in the risk of disease associated with an infectious bite in all individuals rather than complete protection in 50% of individuals. A vaccine with this kind of effect is often referred to as a "leaky vaccine". A consequence of the use of a leaky malaria vaccine is that over time both vaccinated and comparator children will continue to experience malaria episodes and that in high transmission areas following sufficient exposure and extended follow-up, all individuals will experience an infection, regardless of whether they were vaccinated or not. However, this does not mean that vaccination would not provide public health benefit, since the number of episodes could be significantly reduced as well as the risk of complications.

In endemic areas, young children may experience several episodes of malaria per year and gradually acquire immunity against malaria disease through repeated exposure to parasites. Naturally acquired immunity is predominantly directed to the blood stage of the infection, in contrast to the vaccine, which induces protection through an effect on the pre-erythrocytic stage. Vaccine trials are conducted superimposed on this background making long-term vaccine efficacy difficult to disentangle from protection acquired by exposure to infection.

Vaccine efficacy is calculated as VE = 1 - R where R may be a ratio of risks, rates or hazards. All of these methods have been used to license vaccines. The choice of method is dependent upon the data, but it is also important that the output is meaningful to the end user: those making vaccine policy decisions and eventually parents. In this trial, vaccine efficacy is calculated using comparison of risks, rates, or hazards depending on the endpoint. The purpose of this paper is to set out the statistical methods that will be used to estimate the efficacy of RTS, S/AS01. The choice of statistical analysis for endpoints in the RTS, S/AS01 phase III trial is used to illustrate the advantages and disadvantages of each method.

## Trial design

The RTS, S/AS01 pivotal phase III trial is a double-blind (observer-blind), randomized, controlled study conducted in 11 centres located across seven countries in sub-Saharan Africa enrolling children in two age categories: 6-12 weeks and 5-17 months old with a minimum of 6,000 subjects in both age categories up to a maximum of 16,000 children and infants overall. In both age categories, the subjects are randomized to one of the three study groups in a 1:1:1 ratio; (i) RTS/SAS01 given in a 0,1,2-month schedule + a RTS, S/AS01 booster dose at Month 20 (group R3R); (ii) RTS/SAS01 given in a 0,1,2-month schedule + a booster dose of control vaccine at Month 20 (group R3C); (iii) control vaccine given in a 0,1,2-month schedule + a booster dose of control vaccine at Month 20 (group C3C) [1]. Children in the 6-12 week age group receive the primary course of RTS, S/AS01 co-administered with their routine infant immunizations (diphtheria, tetanus, whole cell pertussis, hepatitis B, *Haemophilus influenzae *type B and oral polio vaccines).

The full list of efficacy endpoints is given in Table 1. Case definitions are given in the companion papers including those for clinical malaria and other morbidity endpoints [1] and severe malaria [2].

**Table 1:** Efficacy endpoints

Primary Efficacy Endpoint	Efficacy against clinical malaria when primary immunization starts at 6-12 weeks, or 5-17 months of age Occurrence of cases of malaria meeting the primary case definition for clinical malaria over a period starting 14 days post Dose 3 for 12 months in children aged 6-12 weeks
	
	Occurrence of cases of malaria meeting the primary case definition for clinical malaria over a period starting 14 days post Dose 3 for 12 months in children aged 5-17
**Secondary Efficacy Endpoints**	**Efficacy against severe disease** Occurrence of severe malaria meeting the primary and secondary case definitions
	
	**Efficacy against incident severe anaemia and malaria hospitalization** Occurrence of incident severe anaemia and malaria hospitalization meeting the primary and secondary case definitions
	
	**Duration of efficacy of a primary course** For a primary schedule without a boost, the occurrence of clinical malaria meeting the primary case definition analysed over the time periods starting 14 days post Dose 3 until boost, boost until study end and 14 days post Dose 3 until study end
	
	**Potential added benefit of a booster dose** For a primary schedule with and without a boost, the occurrence of clinical malaria meeting the primary case definition analysed over the time period starting at boost until study end
	
	**Efficacy under different transmission settings** For each site, occurrence of clinical malaria disease meeting the primary case definitions
	
	**Efficacy against secondary case definitions of clinical malaria** Occurrence of clinical malaria disease meeting the secondary case definitions
	
	**Efficacy against prevalence of parasitaemia** Presence of parasitaemia at 18 months and 30 months post Dose 3 and 12 months after the booster dose
	
	**Efficacy against prevalence of moderate and severe anaemia** Presence of moderate and severe anaemia at 18 months and 30 months post Dose 3 and 12 months after the booster dose
	
	**Efficacy against other serious illness** Occurrence of other serious illness meeting the primary and secondary case definitions. Other serious illness is all medical hospitalization, sepsis and pneumonia
	
	**Efficacy against fatal malaria and all-cause mortality** Occurrence of fatal malaria (meeting the case definitions) and all-cause mortality
	
	**Effect on growth** Compare the height/length, weight and mid-upper arm circumference for age z-score
	
	**Gender-specific efficacy** In male and female children, the occurrence of clinical malaria disease meeting the primary case definition

The co-primary endpoints are efficacy against clinical malaria for 12 months post Dose 3 in both the 6-12 weeks and 5-17 months age groups. The analysis of efficacy will be carried out when 6,000 children in an age category have been enrolled and followed up for 12 months post Dose 3. Vaccine efficacy (VE) against first or only episodes will be determined using a standard formula: VE = 100 × (1 - HR)% where HR is the hazard ratio estimated from Cox regression model. Following an episode of malaria, an individual will be removed from the risk set and subjects who are lost to follow-up, who are withdrawn, or who die will be included up to the date of loss, withdrawal or death [3]. Sites are considered as strata, thus allowing for the fact that the temporal pattern of incidence rate in the control group may vary from site to site. The time scale for analysis is the elapsed time since 14 days after dose 3 (for the ATP analysis), and elapsed time after dose 1 (for the ITT analysis), rather than calendar time. This allows all randomized subjects to be included in the risk set at the start of follow-up, and it simplifies analysis of duration of protection. A common case definition will be applied in all sites, chosen to have high specificity in all sites.

The primary analysis will be stratified for study site and unadjusted for other covariates; however, vaccine efficacy estimates adjusted for covariates will also be presented. As there are two co-primary endpoints, 97.5% confidence intervals (CIs) on VE will be calculated, ensuring an overall 2-sided 5% alpha-level. Therefore, p-values lower than 0.025 will be considered significant for the primary endpoints. Assuming at least 5400 evaluable subjects (randomized 2:1), an attack rate in controls of 10/100 per child year at risk (cyr) over the follow-up period from 2 weeks post Dose 3 to 1 year post Dose 3 and a true vaccine efficacy of 30%, the study has 90% power to detect a lower limit of the 97.5% CI around estimated VE above 0%. All secondary endpoints will be evaluated using 95% CIs.

VE against all episodes will be calculated by an incidence rate ratio (IRR) using a negative binomial regression model controlling for interdependence between episodes within the same subject. The 95% CI and p-values on VE estimates, defined as 100 × (1 - IRR)%, will be calculated from this model. Events occurring within 14 days following a malaria episode meeting the malaria case definition under evaluation will not contribute, to minimize counting treatment failures as an episode resulting from a new infection and the total time at risk will be adjusted.

VE against severe malaria will be evaluated when 250 subjects have reported an episode of severe malaria meeting the primary case definition. Recruitment of 250 affected subjects provides 90% power to detect 30% VE with a lower limit of 95% CI above 0%. The primary analysis will compare the pooled RTS, S/AS01 groups versus the control group (R3R + R3C vs C3C). VE against severe malaria will be estimated as 1 - RR where RR is the risk ratio (proportion of subjects reporting severe malaria in the RTS, S/AS01 group over the proportion in the control group) together with 95% CIs. Analysis of all events will be performed in a similar way as for clinical malaria disease. Table 2 summarizes the analytic approach of efficacy applied to each endpoint.

**Table 2:** 

	Endpoint
**Risk ratio**	At least one episode:
Comparison of proportion affected	Severe malaria
	Incident severe anaemia
	Malaria mortality
	All cause mortality
	Prevalent *P. falciparum *infection
	Prevalent anaemia

**Rate ratio**	All episodes:
Negative binomial regression model	Clinical malaria
	Severe malaria
	Malaria hospitalization
	All cause hospitalization
	Sepsis
	Pneumonia

**Hazard Ratio**	First or only episode:
Cox regression model	Clinical malaria

The benefit of the booster dose will be evaluated by the comparison of RTS, S/AS01 recipients receiving a booster dose compared to control booster (R3R vs R3C) over the 12 months following booster dose. Children receiving a primary schedule with or without booster dose will be compared to controls as well (R3R vs C3C and R3C vs C3C).

At 18 and 30 months post primary immunization, a cross sectional survey will sample all participating children to assess vaccine efficacy against prevalent parasitaemia and anaemia. This will be estimated by the prevalence ratio with 95%CI.

The primary efficacy analysis will be performed on the according to protocol (ATP) population over the period starting 14 days post Dose 3. The ATP population includes all subjects meeting the inclusion criteria who received all vaccinations according to protocol and contribute to time at risk starting 14 days post dose 3. The choice to perform the primary analysis on the ATP population reflects the thought that the main outcome is to describe vaccine efficacy for a vaccinated population. However, analyses will also be conducted on a modified intention to treat (ITT) population, which includes all subjects who received at least one dose of vaccine. The ITT population will be analysed by randomization assignment and start time at risk from the day of first vaccination. Subjects randomized but not vaccinated are not under efficacy surveillance.

## Assessment of vaccine efficacy against clinical malaria

Case definitions - malaria is characterized by fever caused by parasitaemia. In malaria endemic regions it is not uncommon for children to carry asymptomatic infections with *P. falciparum*. Fever in these children may be caused by disease other than malaria. In order to improve specificity, cases in this trial are defined as children who are unwell and have presented to the clinic with fever and parasite density above a threshold value [4]. A common parasite density threshold (>5,000/mcl) was chosen to have good specificity in all sites, recognizing that this means sensitivity will vary from site to site. Secondary case definitions include other thresholds [1].

The percentage of children free of malaria by the end of the trial can be compared but efficacy will depend on the level of incidence and the duration of the trial so this measure of efficacy is difficult to compare between trials and it does not reflect the reduction in disease burden due to vaccination. As a primary analysis, vaccine efficacy is estimated by evaluating the hazard ratio of first or only episodes, allowing evaluation of the direct effect of the vaccine. This is in line with the hypothesized mechanism of RTS, S/AS vaccination, namely that rather than providing complete protection to half of the individuals (all or nothing type vaccine), it protects by reducing the risk of disease following an infectious bite by a certain proportion for all individuals. The Cox model allows for different levels of baseline risk across study sites and can include baseline covariates. Under the assumption that vaccine efficacy is not different across study sites and the hazard ratio is constant over time, it is a good method to reduce variability and control for confounding, particularly as data for the primary endpoint will be pooled across multiple transmission settings. Also, analysis on vaccine efficacy by study site is a secondary analyses planned for by the protocol. This methodology is well established and has been broadly used to evaluate previous malaria interventions [5-13] and to license many drugs and vaccines. This method is acceptable to regulatory authorities and WHO as a measure of VE [1,14], although a shortcoming of the vaccine efficacy calculated from hazard ratios could be that it is not intuitively understood.

Vaccine efficacy is also calculated against all episodes of clinical malaria. The methodology used to do this must take into account the fact that the risk in all individuals is not equal; some individuals are more susceptible than others related to host factors such as genetic background, health and behaviour or to the level of exposure to bites by infectious mosquitoes in the community. Moreover, the risk of malaria may not be constant over subsequent episodes. Vaccine efficacy is calculated by comparing incidence rates using a negative binomial regression model allowing for individual heterogeneity arising from non-independence of multiple episodes within the same subject. This model accounts for heterogeneity among individuals and considers non-independent multiple episodes [15,16]. Vaccine efficacy based on a comparison of incidence rates is perceived as a straightforward measure that is meaningful to policy makers and can be used for the evaluation of the economic consequences of vaccine introduction.

## Assessment of vaccine efficacy against severe malaria

Unlike uncomplicated clinical malaria, which is a relatively common disease from which patients usually recover completely with treatment, severe malaria is a rare event associated with a high case fatality rate even in the presence of optimal treatment and repeated episodes in the same individual are uncommon [17]. As such, severe malaria remains an important cause of childhood mortality in sub-Saharan Africa [18,19].

Vaccine efficacy against severe malaria disease will be analysed as a risk ratio, comparing the proportion of children affected by at least one episode of severe malaria in each group. The key advantage of this measure is that it is readily interpretable; the vaccine reduces the risk of severe disease over the specified time frame by a proportion. This methodology is also used for each of the endpoints, which share the same features of being rare and life-threatening (Table 2).

In a secondary analysis, all episodes of severe malaria will be investigated in the same way as all episodes of clinical malaria.

## Assessment of vaccine protection over time

The assessment of the evolution of vaccine efficacy against clinical malaria over time is the most challenging aspect of the analysis; it is not a limitation of the statistical methodology but of the data set itself. Two analytic methods will be used to evaluate the evolution of protection against clinical malaria over the 30-month follow up period. The first will compare the study groups R3C and C3C from the beginning to the end of the study and the second will estimate vaccine efficacy over shorter time periods; from 14 days post dose 3 to 17 months and from 18 months to 30 months. To evaluate duration of protection, analyses of all events is more informative than first or only episodes.

The challenge lies in the interpretation of the results that these analyses will provide because neither methodology is able to disentangle the effects of vaccine induced immunity and naturally acquired protection. In most parts of Africa, children acquire a high level of protection against the severe forms of malaria in early childhood as a result of repeated exposure to blood stage parasites. It is expected that the vaccinees also acquire immunity as a result of exposure to infection. The difficulty is that there is no immunological assay that can tell whether vaccinees have acquired natural immunity at the same rate as controls and allow adjustment for this in the analysis. If natural immunity is acquired at a similar rate in controls and vaccines, then estimates of vaccine efficacy will approximate true vaccine efficacy, but if vaccinees and controls acquire natural immunity at a different rate, estimates will not reflect true vaccine efficacy. Although efficacy estimates based on first or only episodes may not be very informative of duration of protection, the Cox proportional hazards method allows to investigate whether the proportionality of hazards remains constant over time (i.e. that observed vaccine effect remains constant) by examining cumulative incidence plots, Schoenfeld residuals and models with time-dependent covariates. If there is no support from the data that the HR changes over time, one could assume a constant effect over the follow-up. However, the opposite is not true: violating the proportionality of hazards assumption does not necessarily mean waning efficacy. Violating a proportionality of hazards assumption in the presence of declining attack rates may suggest decreasing susceptibility or heterogeneity in exposure rather than waning vaccine efficacy. The approach of breaking the surveillance period into shorter time periods, sometimes described as "resetting the clock", is intuitive. However, the interpretation remains complicated by the fact that the control group is no longer directly comparable in the second time period due to different exposure in the first time period and the concomitant development of natural immunity.

As relevant to policy makers as the evolution of vaccine efficacy over time, is the presentation of results addressing the fundamental hypothesis underlying the development strategy for RTS, S/AS01. The hypothesis is that a partially efficacious vaccine would be of great value in endemic areas, if it was able to be given to young children and provide them with protection against the severe life-threatening forms of the disease until they had acquired natural protection. This can be assessed by presenting an analysis of the number of cases averted by the vaccine over time.

## Covariates

A covariate can be defined as a qualitative factor or a quantitative variable that is expected to have an influence on study endpoint(s) to be analysed. Covariates can be highly variable and may include disease characteristics, social or economic factors and geographic or demographic data [20].

Study centres participating in this trial represent a range of malaria transmission intensities across sub-Saharan Africa, including the intense seasonal transmission of West Africa and perennial transmission in East Africa. Other factors might influence the pattern of malaria disease in a population and may be taken into account as covariates for data analyses. The importance to controlling for heterogeneity in malaria risk to avoid underestimating vaccine efficacy has been highlighted earlier [21].

All the covariates collected in this trial have known associations with malaria risk. Cox regression allows for seasonal variation of malaria incidence and adding study centres as a covariate allows for different levels of baseline risk and different patterns of seasonality. The trial collected a number of potential covariates including age, bed net use [22-24], distance from nearest inpatient/outpatient facility [25], pneumococcal vaccination status [26,27], ethnicity [28,29] and nutritional status [30,31].

## Conclusion

In conclusion, the analytic methods selected to analyse the results of the RTS, S/AS01 phase III clinical trial must be scientifically sound, acceptable to regulatory authorities and meaningful to those responsible for malaria control and public health policy. These needs have been taken into account and resulted in a list of primary and secondary endpoints, each with their appropriate analytic methods. Finally, an understanding of the biological action and full benefit/risk profile of the vaccine will only be gained by looking at the full picture that is painted by the entire range of predefined endpoints and planned analyses.

## Competing interests

ML and AL are employees of GSK Biologicals. AL holds stock options in GSK Biologicals. ML, JA, JW, BM, PB, and AL declare their institution received a grant from MVI for the clinical trial described in this manuscript. ML and AL declare their institution has received grants from MVI for previous clinical trials. PB declares his institution has grants pending from MVI. JA, JW, and PB declare receiving travel funds from MVI for travel related to this clinical trial. JA declares his institution received financial compensation from MVI for administrative support. AM declares no potential conflicts of interest.

## Authors' contributions

All authors contributed to the development of this manuscript through discussions and document review. ML led the writing of the manuscript coordinating the incorporation of all reviewer comments. All authors critically contributed to the discussions on statistical methodology. All authors read and approved the final manuscript.
